# Supplementary material for: Dephasing by optical phonons in GaN defect single-photon emitters
Source: Sci Rep. 2023 May 29;13:8678. doi: 10.1038/s41598-023-35003-z (PMC10227053; doi:10.1038/s41598-023-35003-z)
Supplement: Supplementary file 1 — Supplementary Information. [file 41598_2023_35003_MOESM1_ESM.pdf]

Dephasing by Optical Phonons in GaN Defect Single-Photon Emitters: Supplementary Information

Yifei Geng[1,*], Jialun Luo[2], Len van Deurzen[3], Huili (Grace) Xing[1,4], Debdeep Jena[1,4], Gregory David Fuchs[3], and Farhan Rana[1]

[1]School of Electrical and Computer Engineering, Cornell University, Ithaca, New York 14853, USA.
[2]Department of Physics, Cornell University, Ithaca, New York 14853, USA.
[3]School of Applied and Engineering Physics, Cornell University, Ithaca, New York 14853, USA.
[4]Department of Materials Science and Engineering, Cornell University, Ithaca, New York 14853, USA.
[*]yg474@cornell.edu

Fitting a T^3 power law to the ZPL linewidth data

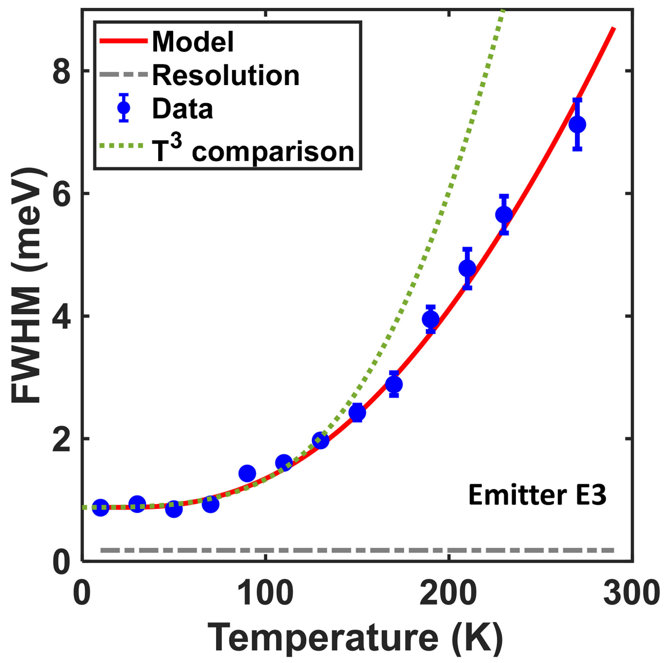

Figure S1. The FWHM linewidth of emitter E3 is plotted as a function of the temperature. The solid line is the fit to the data using the theoretical model discussed in the main text. The dotted line shows an attempt to fit the data with a model in which the temperature dependence of the linewidth is proportional to T^3.

Fig. S1 shows an attempt to fit the ZPL FWHM linewidth data of emitter E3 with a model in which the temperature dependence of the linewidth f_L of the Lorentzian, determined by the dephasing rate γ, is CT^3, where C is a constant. If the value of C is adjusted to fit the data well at low temperatures then the CT^3 model predicts an increase in the linewidth with the temperature that is much steeper than the data, as shown in Fig. S1. It is obvious from this that dephasing mechanisms that give T^5 or T^7 temperature dependence will also not agree with our data.

Deriving the effective hamiltonian for optical phonon induced dephasing

The effective Hamiltonian for optical phonon induced dephasing given in Eq.6 of the main text can be obtained from the electron-optical phonon interaction term in the Hamiltonian given in Eq.5 using the scheme described here. Consider the

following Hamiltonian,

$$H = H_o + H_i \tag{1}$$

where H_i represents a perturbation (in the model presented in this work, H_i would be the electron-optical phonon interaction term in the Hamiltonian in Eq.5). The matrix elements $T_{fi} = \langle f|H^{eff}|i\rangle$ of the T-matrix between any initial and final states, $|i\rangle$ and $|f\rangle$ with respective energies E_i and E_f, can be expressed as a power series in the perturbation H_i[1],

$$H^{eff} = H_i + H_i \frac{1}{E_i - H_o} H_i + H_i \frac{1}{E_i - H_o} H_i \frac{1}{E_i - H_o} H_i + \dots \tag{2}$$

The effective Hamiltonian in Eq.6 corresponds to the second term in the series above as this term captures the elastic Raman-like process, depicted in Fig. 6(a), responsible for dephasing in our proposed model.

References

1. Roman, P. *Advanced Quantum Theory* (Addison-Wesley Publishing Company, 1965), 1st edn.
